# Charting a new course with plasmon-mediated chemistry

**DOI:** 10.1038/s42004-024-01340-x

**Published:** 2024-12-17

**Authors:** Christa L. Brosseau, Emiliano Cortés

**Affiliations:** 1 Department of Chemistry, Saint Mary’s University, Halifax, B3H 3C3 Nova Scotia Canada; 2 Nanoinstitute Munich, Faculty of Physics, Ludwig-Maximilians-Universität München, Munich, 80539 Germany

**Keywords:** Surface plasmon resonance, Nanophotonics and plasmonics

## Abstract

*Communications Chemistry* is pleased to introduce a Collection of articles on the topic of plasmon-mediated chemistry. Here, the Guest Editors discuss different themes within and look towards the future of the field.

## Introduction

The interaction of light with certain nanoscale metals, most notably the coinage metals, gives rise to unique optical and electronic effects. These properties are largely a result of the generation of surface plasmons, which represent a quantized form of plasma energy. Surface plasmons consequently give rise to several important phenomena, including intense electromagnetic fields at the surface, allowing these metal structures to act as nanoantennas and nanowaveguides. These electric fields are also exploited in surface-enhanced Raman spectroscopy (SERS) and localized surface plasmon resonance (LSPR) sensing. Recently, it has been discovered that surface plasmons also give rise to hot charge carriers (hot electrons, hot holes) arising from Landau damping of the plasmon^1^. These hot charge carriers can drive surface chemistry in an entirely new way. Areas that explore photocatalytic reactions using plasmonic nanomaterials are rapidly expanding, with potential advantages that include low energy input, high selectivity, and mild reaction conditions. Despite the potential promise, challenges remain. The ultrafast relaxation of these hot carriers, generally on the fs timescale, has created a bottleneck in the field in terms of application, and a solid fundamental understanding of these entities is lacking. In this Collection on plasmon-mediated chemistry, we have gathered publications that focus on a deeper mechanistic understanding of these processes and their varied contributions, highlighting new materials and applications in the field.

## Mechanistic insight

Plasmon-mediated chemical and electrochemical reactions are increasingly reported, but mechanistic debate remains. It is generally accepted that these mechanisms include i) optical rectification ii) hot charge carrier generation and contribution, and iii) heat generation and conduction. These varying contributions to the observed non-trivial reaction rate enhancements and reaction selectivity that have been reported can be challenging to untangle. Al-Amin et al. explored the photothermal heating effect in plasmon-assisted electrochemistry to quantify and describe this contribution in the larger context of plasmon-mediated chemistry^2^. In this work, the authors revealed the role of temperature gradients in plasmon-assisted chemistry and detailed a simple control experiment to account for this effect. In particular, the authors noted that under illumination, the electrode surface reached an elevated, steady-state temperature while the surrounding solvent quickly dissipated the heat. The resulting temperature gradient induces free convection, contributing to a decreased diffusion layer thickness and an increased current density. Notably, this study found that photothermal heating is a primary contributor to the enhancement observed in plasmon-assisted electrochemistry. This is not the case for the system studied by Dutta el al, in which energetic hot-carriers seem to dominate the reactivity of N-methyl-4-sulfanylbenzamide (NMSB) at nanocavities of gold and silver nanoparticle aggregates. By in-situ SERS monitoring and supported by dissociative electron attachment (DEA) studies with the gas phase molecule, a plasmonic hot electron-mediated conversion of NMSB to p-mercaptobenzamide and p-mercaptobenzonitrile has been proposed by the authors when exploring their plasmonic nanoparticles aggregates^3^. Finally, Coane et al. used state-of-the-art hybrid ab initio / electromagnetic modeling to provide a comprehensive theoretical analysis of tip-enhanced electron energy transfer, providing a framework for a nanostructure–donor–acceptor system^4^. This model is a useful starting point for exploring the role of plasmonic nanostructures in tailoring energy flow across molecules that exist in complex multichromophoric architectures. These contributions highlight the interesting and complex nature of plasmon-mediated chemistry and provide pathways for future advancement and understanding.

## New materials

With the emergence of plasmon-mediated chemistry, there has been a growing interest in new materials that can exploit the potential advantages of this field while also reducing the inherent limitations. Ramachandran et al. studied hot carriers originating from intra- and interband transitions in Au-Ag alloy nanoparticles using an atomistic modeling approach^5^. Interestingly, the authors note that alloy composition was an important tuning parameter for hot carriers — if the gold fraction is high, energetic holes and “cold” electrons are generated while larger silver fractions produce energetic electrons and “cold” holes. Clearly, the alloy composition is important for tailoring the reactivity of nanoparticle-based plasmonic materials. Murastov et al. explored the emergent area of plasmonic nanoparticles coupled with 2D materials^6^. In particular, the authors introduced an approach for edge-specific nanoparticle decoration of WSe_2_ nanoribbons via light-assisted reduction of silver and the merging of silver seeds. The authors postulate that such edge-decorated nanoribbons could have potential applications in plasmonics. These works highlight how tunable, nanoengineered plasmonic materials may advance our understanding of plasmon-mediated chemistry, leading to cutting-edge applications.

## Emergent applications

As collective understanding of plasmon-mediated chemistry evolves, new applications of this field are continually demonstrated. Becerril-Castro et al. highlight the development of UV-vis-NIR light filters using plasmonic nanoparticles and gelatin, a novel nanocomposite material^7^. Taking advantage of the tunable LSPR of the metal nanoparticles provided an avenue for high control over the optical filtering properties. Such optical filters could potentially be biodegradable and biocompatible. Dey et al. explored a plasmonic nanohybrid system, NiO/Au/Re(*phen-NH*_*2*_)(CO)_3_Cl, for direct photoelectroreduction of CO_2_^8^. In this work, the reduction of CO_2_ to form CO was confirmed through photoelectrocatalytic experiments, complemented by mass spectrometry and in situ FTIR. The low thermal stability of the Re complex provided substantial support for the hypothesis that hot electrons were primarily responsible for the observed photocatalytic behavior, as opposed to thermal effects. Libánská et al. explored the combination of surface plasmon resonance (SPR), capillary electrophoresis, and NMR spectroscopy to characterize the pH-triggered release of covalently bound drugs from polymer nanocarriers^9^. Of these techniques, SPR was found to be the most promising for following drug release kinetics and additionally employed flow-through conditions, which is clinically relevant. Ozaki et al. used site-specific mineralization based on artificial peptides and DNA to control the composition of gold-titania nanocomposites^10^. These tailored nanocomposites responded to visible light excitation with potential plasmonic application. This site-specific precipitation strategy is an important advancement for producing multimetallic catalysts and functional materials. Finally, Sood and co-workers developed a one-step colloidal synthesis method for creating PEGylated semi-shells (SS), such as nano-bowls, with unique plasmonic properties due to their asymmetric shape^11^. These SSs exhibit strong localized surface plasmon resonance in the near-infrared region and demonstrate significant photothermal efficiency (~37%) under 808 nm laser irradiation. In preclinical trials, the PEGylated SSs achieved complete remission of breast tumors in mice through photothermal therapy, with a 75% survival rate over 90 days, showcasing their potential in advanced cancer treatment.

## Future outlook

More fundamental studies are needed to unravel the complex mechanisms at play and to help develop strategies to overcome the bottlenecks that currently limit the use of hot charge carriers in reactions of interest. Jin and Higaki provide an important commentary for the field, which identifies and discusses remaining open questions in nanoscience in general, and for plasmonics more specifically^12^. The authors note that the recent success of atomically-precise nanochemistry has led to significant insight, providing answers to decades-old fundamental questions. Perhaps led by these advancements, a better understanding is needed to fully elucidate the role that nanoparticle shape and surface ligands play as one moves from the nanoscale to the bulk. Additionally, such advancements may help clarify the nature of nanoparticle-based catalysis, helping to unravel mechanistic steps and contributing towards next-generation catalyst design. The future for plasmon-mediated chemistry is very bright. As new avenues open for mechanistic discovery, novel materials, and advanced applications, this new pathway for chemical reactivity is helping scientists chart a future course of discovery.
